# Mycolactone induces cell death by SETD1B-dependent degradation of glutathione

**DOI:** 10.1371/journal.pntd.0008709

**Published:** 2020-10-02

**Authors:** Birgit Förster, Caroline Demangel, Thorsten Thye

**Affiliations:** 1 Bernhard Nocht Institute for Tropical Medicine, Dept. Infectious Disease Epidemiology, Hamburg, Germany; 2 Immunobiology Infection Unit, Institut Pasteur, INSERM U1221, Paris, France; NIH-NIRT-ICER, INDIA

## Abstract

*Mycobacterium ulcerans* is a human pathogen that causes a necrotizing skin disease known as Buruli ulcer. Necrosis of infected skin is driven by bacterial production of mycolactone, a diffusible exotoxin targeting the host translocon (Sec61). By blocking Sec61, mycolactone prevents the transport of nascent secretory proteins into the endoplasmic reticulum of host cells. This triggers pro-apoptotic stress responses partially depending on activation of the ATF4 transcription factor. To gain further insight into the molecular pathways mediating the cytotoxic effects of mycolactone we conducted the first haploid genetic screen with the *M*. *ulcerans* toxin in KBM-7 cells. This approach allowed us to identify the histone methyltransferase SETD1B as a novel mediator of mycolactone-induced cell death. CRISPR/Cas9-based inactivation of *SETD1B* rendered cells resistant to lethal doses of the toxin, highlighting the critical importance of this gene’s expression. To understand how SETD1B contributes to mycolactone cytotoxicity, we compared the transcriptomes of wild-type (WT) and *SETD1B* knockout KBM-7 cells upon exposure to the toxin. While ATF4 effectors were upregulated by mycolactone in both WT and *SETD1B* knockout cells, mycolactone selectively induced the expression of pro-apoptotic genes in WT cells. Among those genes we identified *CHAC1*, which codes for a major glutathione (GSH)-degrading enzyme, and whose strong upregulation in mycolactone-treated WT cells correlated with a marked reduction in GSH protein level. Moreover, GSH supplementation conferred cells with substantial protection against the toxic effects of mycolactone. Our data thus identify SETD1B/CHAC1/GSH as a novel, epigenetic mechanism connecting Sec61 blockade with apoptotic cell death. They suggest that GSH-based treatments might have the capacity to limit skin necrosis in Buruli ulcer.

## Author summary

The human pathogen *Mycobacterium ulcerans* causes a necrotizing skin disease known as Buruli ulcer. The major toxin of the mycobacteria, mycolactone, prevents the transport of secretory proteins into the endoplasmic reticulum, and thereby triggers a deadly stress response. We conducted the first haploid genetic screen to identify host factors with impact on mycolactone toxicity. This enabled us to identify the histone methyltransferase SETD1B as a novel mediator of mycolactone-induced cell death. RNA analyses of wild-type cells and resistant *SETD1B* knockout cells treated with mycolactone then showed a selective induction of genes implicated in programmed cell-death only in wild-type cells. This was accompanied by a marked reduction of the antioxidant glutathione, which might cause the mycolactone induced cell death.

## Introduction

Infection with *Mycobacterium ulcerans* causes Buruli ulcer, a skin disease characterized by chronic necrotizing lesions. The pathology of Buruli ulcer is due to bacterial expression of a diffusible toxin called mycolactone [1–3]. In addition to exerting systemic immunosuppression, mycolactone provokes apoptotic cell death in infected skin, leading to the development of ulcers [1, 2]. The intracellular target of mycolactone has been identified as the translocon Sec61 [4–7]. Blockade of this protein complex prevents the import of membrane-anchored and secreted proteins from the cytosol into the endoplasmic reticulum (ER), leading to accumulation of misfolded proteins in the two compartments [1, 8]. This triggers an integrated stress response (ISR) and an unfolded protein response (UPR) [8, 9] both activating the translation factor 2α (EIF2α)[8]. A target gene of EIF2α is *ATF4*, which enhances the expression of genes regulating resistance to oxidative stress and amino acid deprivation [10]. Sustained overactivation of ATF4 however can lead to apoptosis in affected cells [11]. While there is robust evidence that mycolactone triggers programmed cell death via the ATF4/CHOP/Bim signaling pathway, silencing ATF4 only conferred partial protection against the toxin [8, 9, 12]. To gain further insight into the mechanisms underpinning mycolactone-driven apoptosis, we conducted a forward genetic screen using mutagenized haploid KBM-7 cells treated with lethal doses of mycolactone. The approach is based on random gene knockouts of the haploid KBM-7 cells which was derived from a patient with CML.

Genome analyses of KBM-7 cells surviving the effects of mycolactone allowed us to identify SETD1B as a novel and critical mediator of cell resistance to the toxin. In addition, transcriptome analyses of mycolactone’s impact on cells deficient or not for SETD1B expression highlighted differentially expressed genes, providing an explanation for how cell death is triggered by mycolactone.

## Results

### Haploid screen

**Fig 1:**
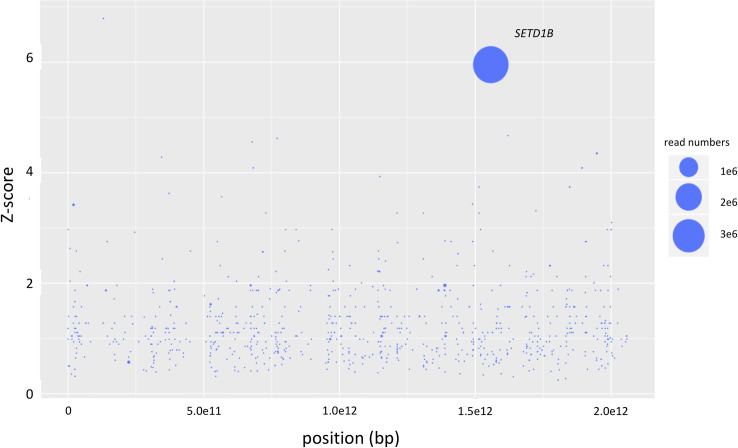
Results of the haploid genetic screen with mycolactone. Genes with inactivating mutations in mycolactone-selected samples are depicted. The size of the circles reflects the number of reads aligning to a specific gene. Genes are ranked on the x-axis according to their chromosomal position and along the y-axis according to the significance of the enrichment of gene-trap insertions in the indicated gene compared to an unselected control dataset. Genes with unequal distribution of reads between selected and un-selected samples having a Fisher Z-score p-value lower than 0.01 as calculated by the HASAPPY software are labeled.

**Fig 2:**
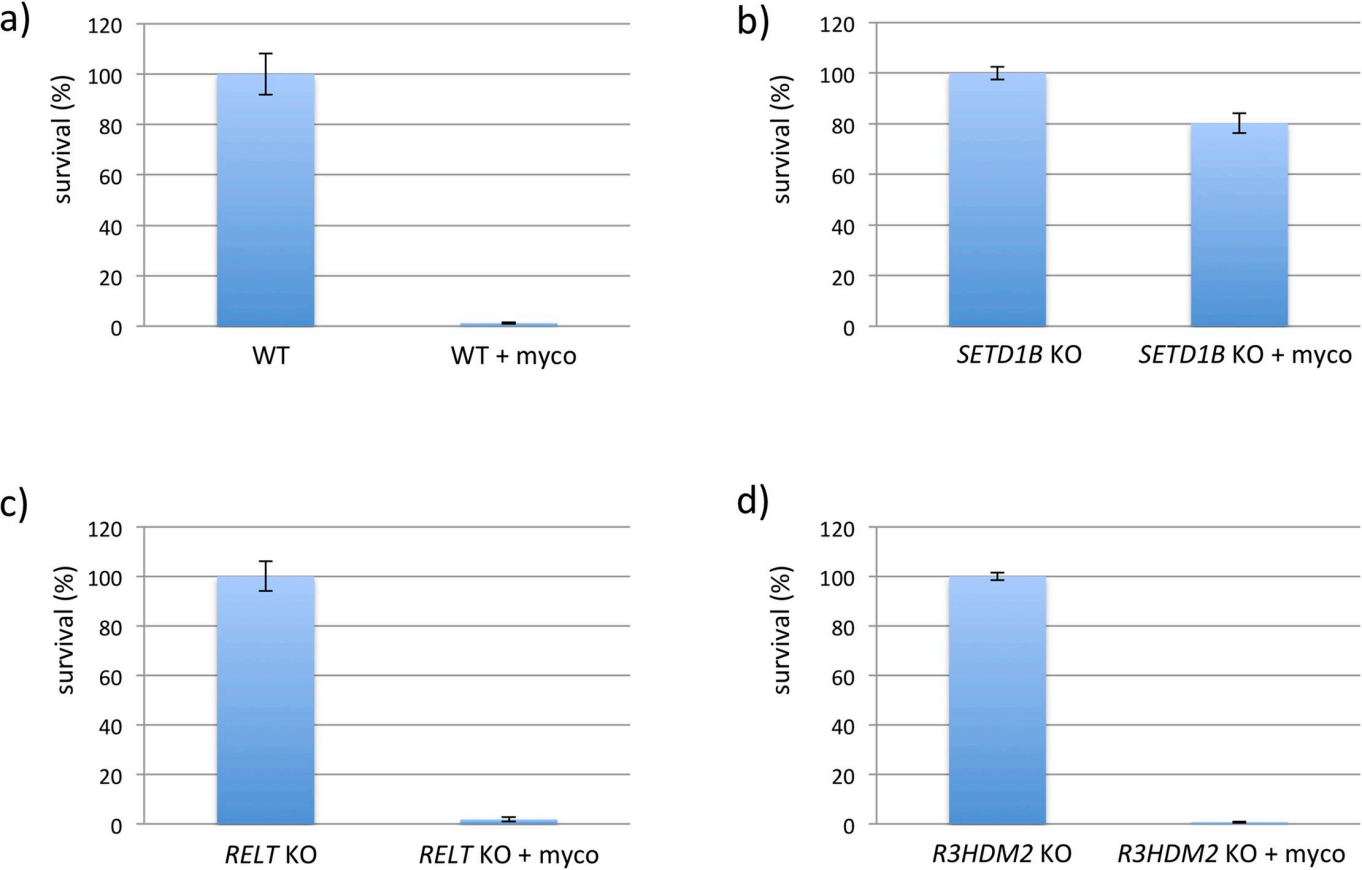
CRISPR/Cas KO clones challenged with mycolactone. Single KBM-7 knockout clones (20.000 cells) of *SETD1B*, *RELT* and *R3HDM2* generated with CRISPR/Cas were treated with a lethal dose of 10 nM mycolactone for 6 d. Surviving cells were counted by FACS analyses based on the GFP fluorescence. Experiments were performed in triplicates.

Mutagenized haploid KBM-7 cells were grown in the presence of a lethal dose of 20nM mycolactone for 2–3 weeks in 96 well plates. Cells of resistant clones identified in two wells out of three 96 well plates were harvested when a density of 10^7^ cells was reached. After DNA isolation and next-generation sequencing analysis on a MiSeq the resulting sequencing reads were mapped against the human reference genome hg19 with Bowtie 2.0. Analyses of the number, frequency and positions of disruptive insertions per gene with the HaSAPPy software yielded a clone (clone 1) with massively increased read counts in the genes *SETD1B*, *R3HDM2* and *RELT* (S1 Table). Only insertions of *SETD1B* were found to be in the direction of the gene’s reading frame, and were found differentially distributed between mutagenized cells treated or not treated with mycolactone (Fig 1). To test whether the insertions in the three genes occur in the same cell we performed single cell dilution to obtain clonal populations. Sequencing analyses confirmed that all three insertions occur in a single cell. We generated knockout (KO) cell lines for each of the three genes to test the independent contribution of SETD1B, R3HDM2 or RELT to the resistance phenotype. Only cells with defective *SETD1B* expression were protected from lethal doses of mycolactone (Fig 2), highlighting the critical importance of this gene in cell resistance to the toxin.

Analysis of the second clone (clone 2) yielded insertions with high read numbers at different positions (S2 Table). Notably, the highest read count corresponded to an insertion located upstream of the *ASH2 like histone lysine methyltransferase complex subunit* (*ASH2L*) gene, which is like SETD1B a component of the ‘Complex of Proteins Associated with Set1’ (COMPASS) complex. This insertion disrupted the binding motif of transcription factor MAFK at chromosomal position chr8:37940930, which is known to interact with ASH2L [13]. In addition, the integration site is known to influence the transcriptional activity of the *ASH2L* gene identified by previous eQTL experiments (SNP rs75551965; http://www.eqtlgen.org/cis-eqtls.html).

In all, both clones identified by our haploid screen pointed to epigenetic resistance mechanisms involving members of the COMPASS complex. Given the critical importance of *SETD1B* in cell resistance to the toxin, we focused the next experiments on this gene.

### Transcriptome profiling of WT and resistant SETD1B KO KBM-7 cells

To address the impact of SETD1B on mycolactone-induced cell death we performed differential transcriptome analysis with RNA isolated from WT KBM-7 cells and *SETD1B* defective cells grown with or without the toxin. RNA was transcribed to cDNA, and next-generation sequencing analyses generated ~31 millions reads per experiment.

**Fig 3:**
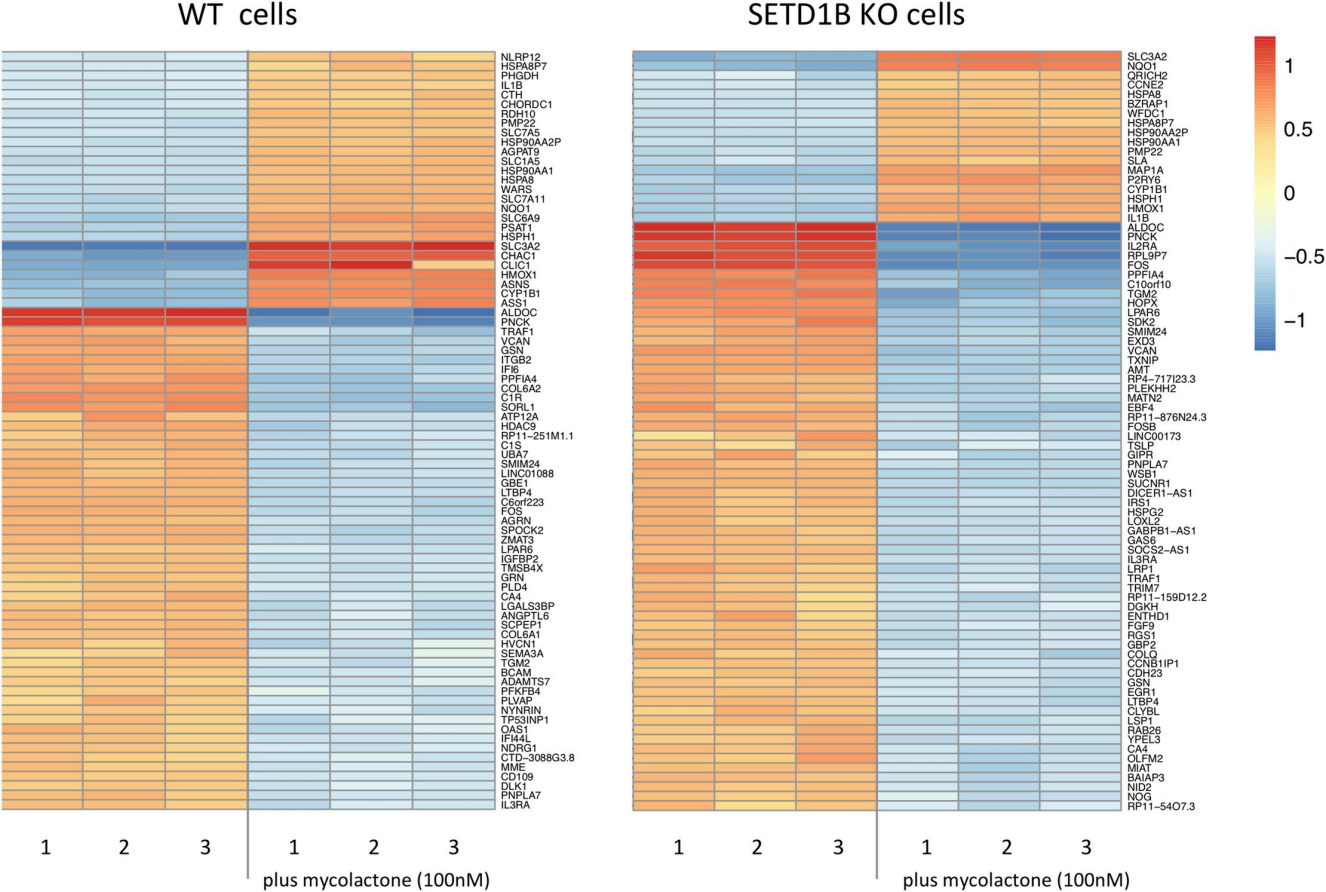
Heatmap of up- and down- regulated transcripts in WT and KO cells with and without mycolactone. The transcriptomic profiles from WT and KO KBM-7 cells (5x10^6^ cells per experiment), treated and not treated with 100nM mycolactone for 24h were determined by analysis with Deseq2 software. Shown are the 60 most strongly differentially regulated genes between treated and non-treated WT cells (left) and treated and non-treated KO cells (right). Color intensity reflects the normalized read counts, where blue mark downregulated and red mark upregulated transcripts.

Differential analysis yielded 184 upregulated genes (> 1 log fold change logFC]) between treated and non-treated WT cells, and 487 genes between treated and non-treated *SETD1B* KO cells (S3 Table). Mycolactone treatment also decreased the expression (> 1 logFC) of 615 genes in WT cells and 1116 genes in the SETD1B KO cells (S3 Table). Consistent with previous work [9], the most upregulated transcripts in mycolactone-treated WT KBM-7 cells as determined by gene ontology (GO) terms were involved in the UPR or mitochondrial stress response (S4 Table). In contrast, GO analysis of mycolactone-treated SETD1B KO cells indicated an enrichment in transcripts important for the proteasome and its accessory complex with log fold changes > 1 (S4 Table, Fig 3).

**Fig 4:**
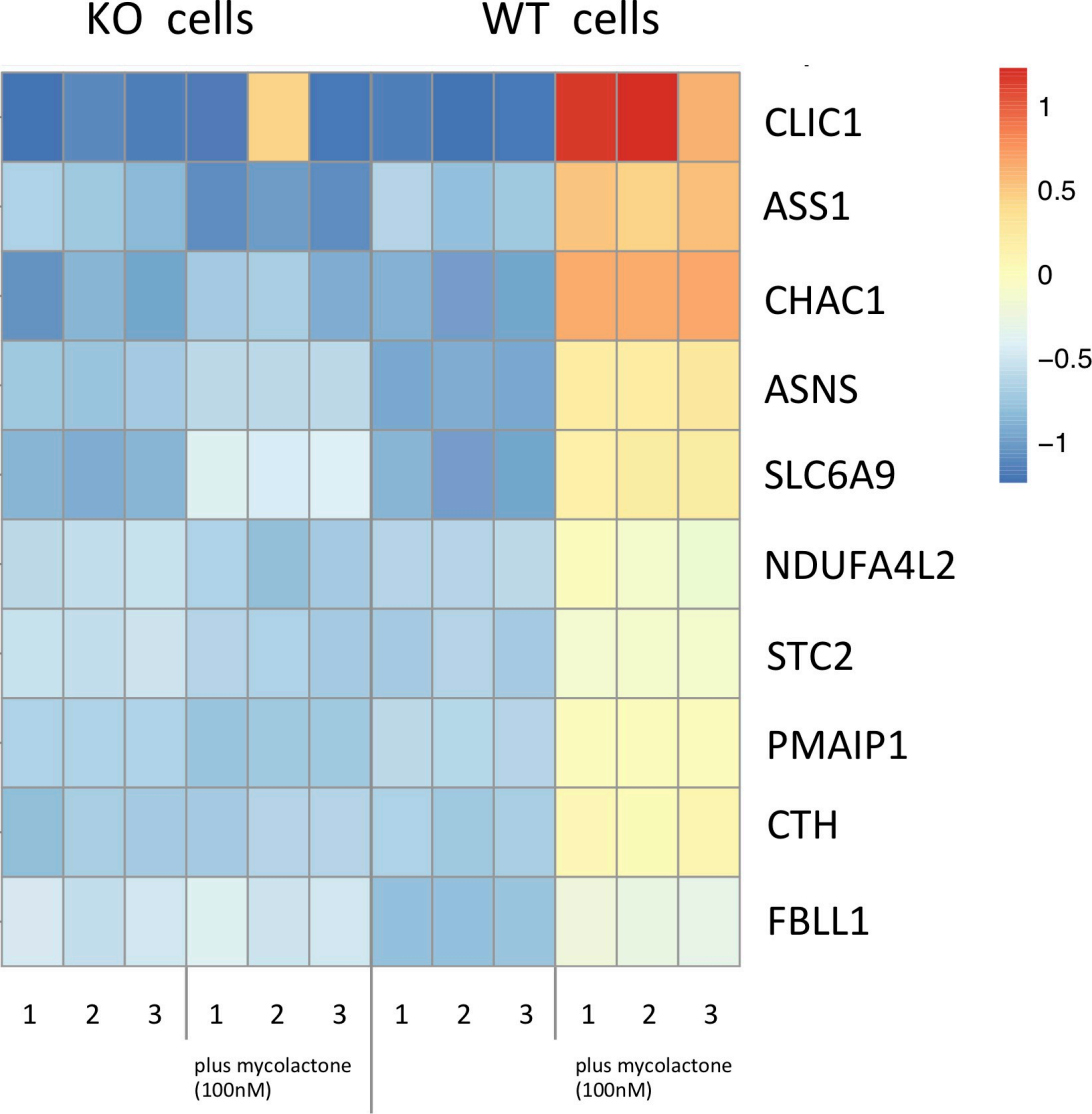
Heatmap of transcripts upregulated only in WT cells after mycolactone incubation. Transcripts found in the WT and KO KBM-7 cells (5x10^6^ cells per experiment), treated and not treated with 100mM mycolactone for 24h. Shown are the 10 most strongly differentially regulated genes, which are only upregulated in WT cells and not in the *SETD1B* KO cells after incubation with the toxin.

To identify resistance mechanisms involving SETD1B, we next focused our analysis on genes that were significantly upregulated by mycolactone in WT cells, compared to SETD1B KO cells. In addition, as it is known that mycolactone triggers apoptosis via the transcription factor ATF4 [8], we placed special emphasis on ATF4 target genes implicated in programmed cell death. Transcriptomic profiling revealed that *ATF4* itself was selectively upregulated in WT cells (WT logFC 0.7, Padj 1.2xE-159 vs *SETD1B* KO logFC 0.0, Padj 0.9), implying that SETD1B regulates ATF4-dependent cell death programs. Among ATF4 target genes, those that were the most strongly upregulated by mycolactone in WT cells compared to *SETD1B* KO cells were the *gamma-glutamylcyclotransferase 1* (*CHAC1*), the *chloride intracellular channel protein 1* (*CLIC1*) and the *phorbol-12-myristate-13-acetate-induced protein 1 PMAIP1* (*NOXA*) gene (Fig 4). Notably, CHAC1 digests glutathione (GSH) into the peptide Cys-Gly and 5-oxoproline and decreased GSH levels have been linked to apoptotic cell death [14]. Mycolactone-treated WT cells appear to overexpress *CLIC1*, which codes for a regulator of GSH synthesis (Fig 4) [15, 16]. PMAIP1 is a BH3 only pro-apoptotic protein that induces apoptosis by interfering with the outer mitochondrial membrane [17]. While other genes were differentially regulated between WT and KO cells, we could not deduce their contribution to mycolactone-induced cell death from their assumed function. Since CHAC1 and CLIC1 are both connected to GSH levels, we further analyzed their contribution to mycolactone toxicity.

**Fig 5:**
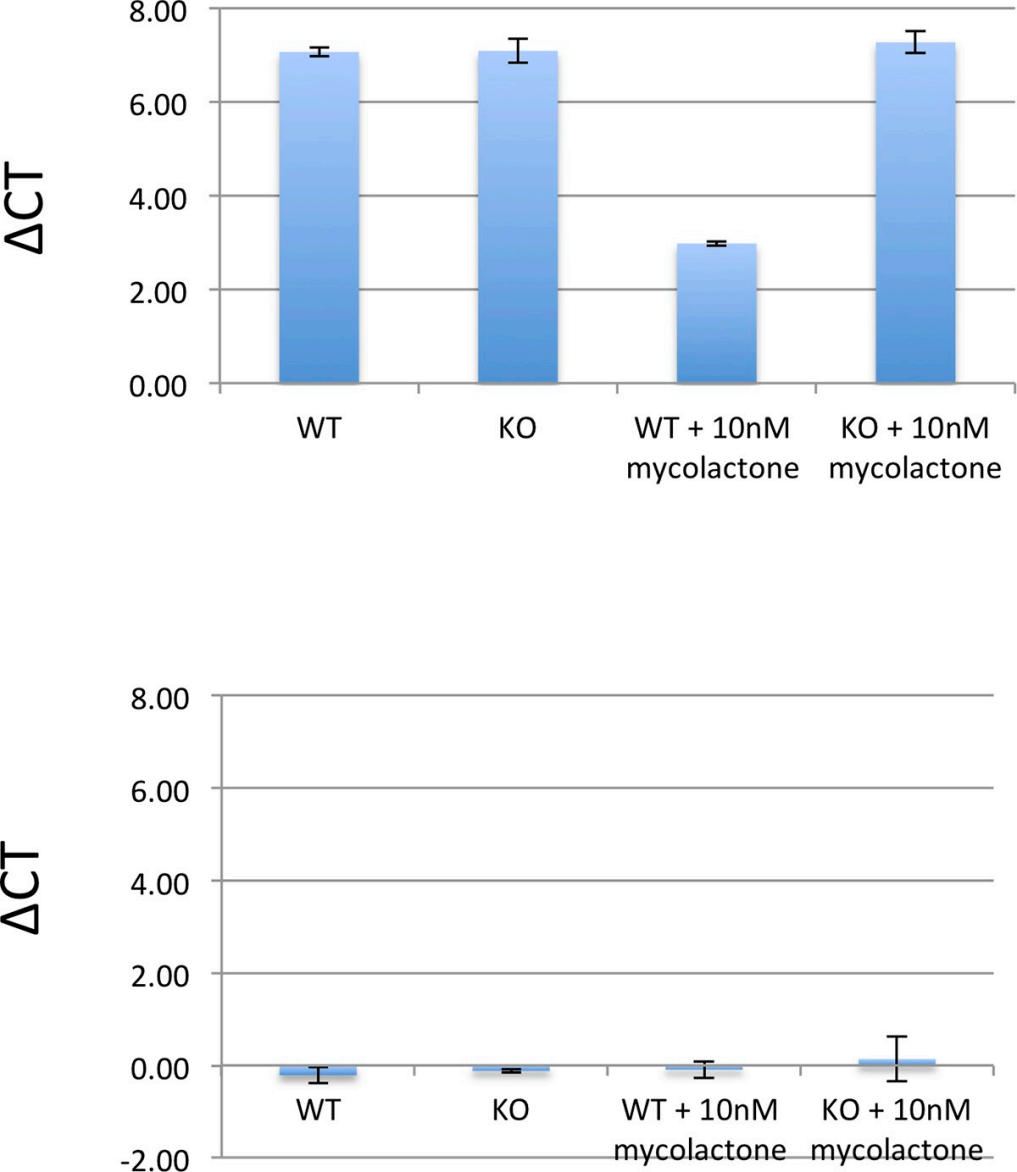
RT-PCR results of *CHAC1* and *CLIC1*. Depicted are ΔCT values of RT-PCR performed with primers for *CHAC1* and *CLIC1* corrected for the household gene *PPA1*. logFC values were calculated by comparing ΔCT values *CHAC1*/*CLIC1* between mycolactone treated WT and resistant KO cells.

To further validate the results obtained from the RNA-Seq analysis we performed RT-PCR analysis for the genes *CHAC1* and *CLIC1*. We found a similar picture of the differentially expressed CHAC1 genes with a logFC of 1.3 (p 6E-4) between WT and SETD1B KO treated with mycolactone cells but were not able to confirm the results of the CLIC1 gene (logFC 0.05; p 0.51) (Fig 5).

### Inhibition of pathways inducing programmed cell death

**Fig 6:**
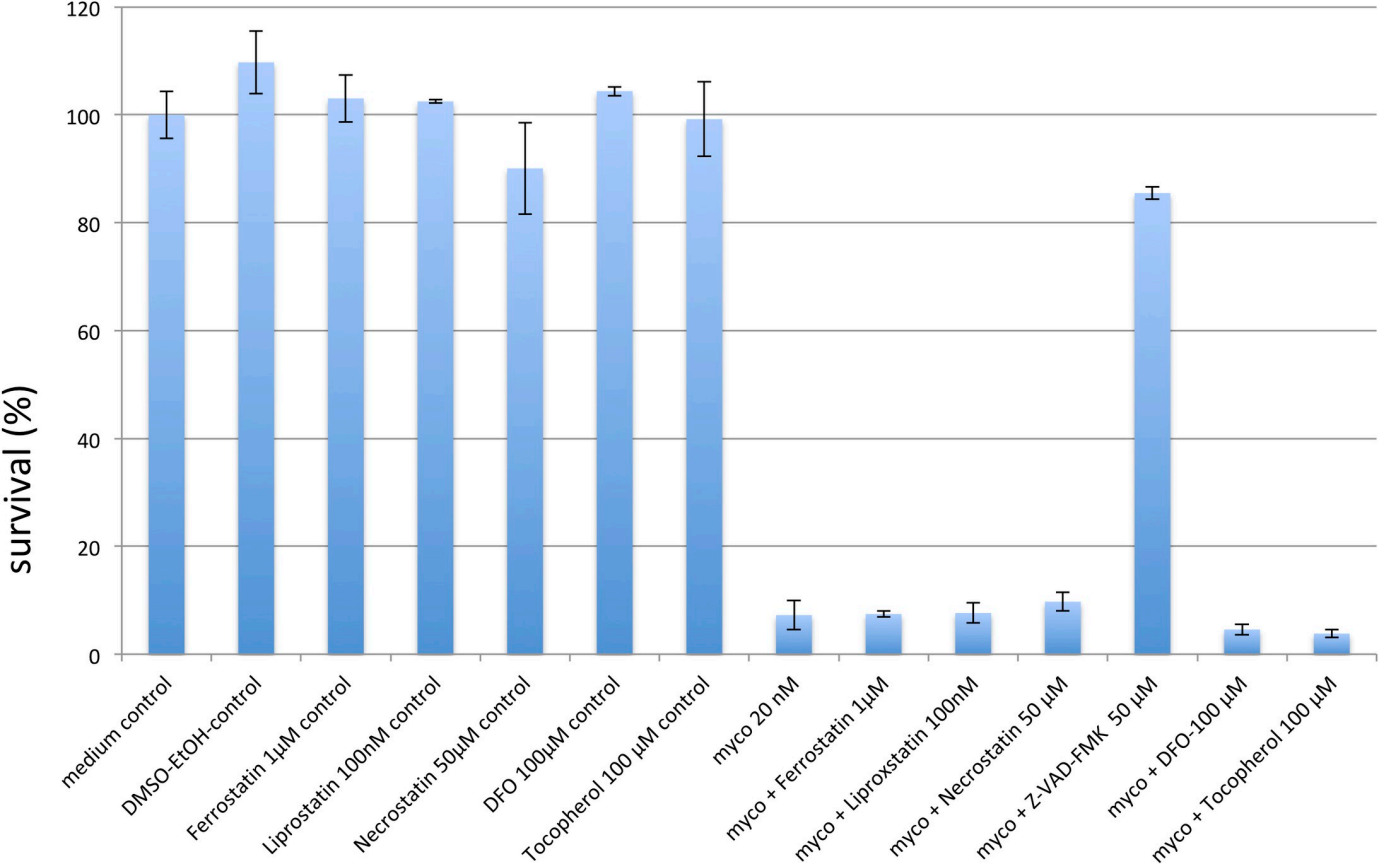
Programmed cell death inhibitors. Depicted are survival rates of WT KBM-7 cells (20.000 cells) challenged with 20 nM mycolactone for 24h after pre-incubation with inhibitors of the programmed cell death pathways of apoptosis (zVAD-FMK, 50μM), necroptosis (necrostatin, 50μM) and ferroptosis (ferrostatin, 1μM; liproxstatin 100nM), and the anti-oxidants deferoxamine (DFO, 100μM) and tocopherol (100μM), or vehicle control (DMSO-EtOH). Data are mean survival rates, compared to medium control (Ctrl). Experiments were performed in duplicate.

Mycolactone was found to induce apoptosis in several cell lines originating from different tissues. However, as we found CHAC1 to be differentially regulated between WT and KO cells and the protein has been implicated in ferroptosis, a cell death pathway different from apoptosis, we tested inhibitors of apoptosis, necroptosis and ferroptosis in WT cells incubated with mycolactone. Consistent with previous studies [12] the pan-caspase inhibitor zVAD-FMK effectively reduced the toxicity of mycolactone (Fig 6). In contrast, inhibitors of ferroptosis or necroptosis did not reduce mycolactone-induced cell death. This includes deferoxamine and the vitamin E derivate α-tocopherol, previously reported to limit the cytotoxic action of mycolactone [18]. Together, these results suggest that CHAC1-associated ferroptosis does not contribute to mycolactone toxicity.

### Glutathione (GSH) and N-acetylcysteine (NAC) supplementation

**Fig 7:**
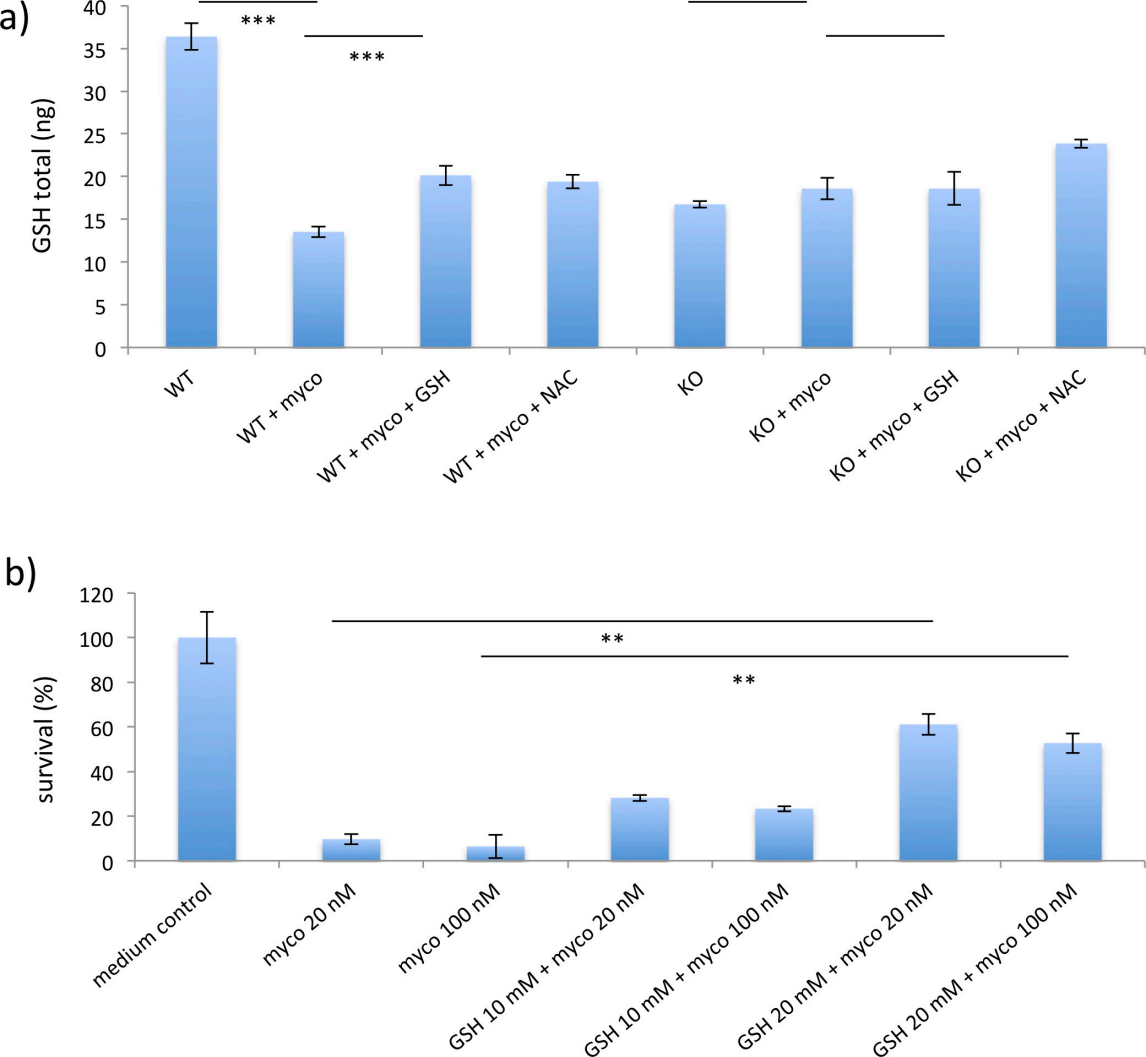
GSH levels in mycolactone treated cells. **a**) GSH levels of WT KBM-7 cells and WT cells treated with 100nM mycolactone for 24h, (n = 3) are shown in addition to the GSH levels of *SETD1B* KO KBM-7 cells with and without 100nM mycolactone for 24h, (n = 3). In the same graph the GSH concentration of WT cells pre-incubated with 10mM GSH or 10mM NAC, which were then challenged with 100nM mycolactone, (n = 3) are presented. **b)** Survival rates of WT cells in the presence of 20 or 100nM mycolactone incubated with 10 and 20nM GSH. Data are representative of 3 independent experiments. For all analyses 20.000 cells were applied. Statistical significance are annotated as *P < 0.05, **P < 0.01 and ***P < 0.001.

CHAC1 is known to degrade GSH and thereby reduce the ability of cells to buffer toxic reactive oxygen species (ROS) [19]. Since CHAC1 was upregulated by mycolactone in WT, but not SETD1B KO cells we measured total intracellular GSH levels in WT and KO cells with and without mycolactone. We found basal levels of GSH lower in KO cells, compared to WT cells (Fig 7A). In addition, mycolactone treatment only induced a reduction in GSH levels in WT cells and not in SETD1B KO cells, which might be caused by the increased CHAC1 expression seen in the WT cells. (Fig 7A and 7B). We then incubated WT and KO cells with either GSH or NAC to fill the intracellular GSH pools. Pre-incubation with GSH and NAC increased cellular GSH levels in mycolactone-treated WT cells and KO cells (Fig 7A). To test whether supplementation with GSH had an impact on survival of cells in the presence of mycolactone WT cells were incubated with GSH for 2–3 h. Pre-incubated WT cells were found to be significantly more resistant to the toxin compared to cells not pre-incubated with GSH (Fig 7B).

## Discussion

With the first genetic screen for mycolactone mediated cytotoxicity we were able to identify the histone methyltransferase SETD1B as an essential element in toxin induced cell death. SETD1B is a member of the COMPASS family of proteins known to enhance gene expression by methylating enhancer or promoter regions [20]. The protein has been found in discrete subnuclear distributions indicating that distinct sets of genes might be regulated [21]. Thus far, conditional knockout analyses in mice suggest that SETD1B plays a role in the development and differentiation of hematopoietic cells [22]. Other studies performed with mouse cell lines indicate that Sod2, Nfkbia, and iNOS were also regulated by Setd1b [23, 24]. Studies with human cell lines found SETD1B to be implicated in the toxic effects of the ferroptosis inducer ML162, and the arginine/proline-rich dipeptide-repeat proteins [25, 26]. Overall it appears that SETD1B is crucial for the development of hematopoietic cells, but further effects and functions of the methyltransferase exists, and become only visible in a context dependent manner [21]. Our data suggest that SETD1B participates in the stress responses triggered by Sec61 blockade [9]. We also found support for another COMPASS related protein, ASH2L, to be implicated in the cytotoxic effects of mycolactone. A viral insertion was found in an ASH2L regulatory element upstream of the gene containing a binding site of the transcription factor MAFK, known to interact with ASH2L [13]. The identification of two constituents of the methyltransferase-activating complex through the haploid screen, in addition to *SETD1B* knock-out experiments clearly underline the importance of the SETD1B bearing COMPASS complex in transmission of the cytotoxic signal.

To investigate the role of SETD1B in induction of the apoptotic signal, and to identify the pathways leading to cell death transcriptome analysis of RNA obtained from WT and KO cells treated or not with the bacterial toxin were performed. The generation and identification of mycolactone resistant cells enabled us to directly compare transcript levels between susceptible WT cells and resistant KO cells, and identify genes which are central for apoptotic cell death triggered by the toxin. Among the transcripts upregulated only in the WT cells after incubation with a lethal dose of mycolactone we found the pro-apoptotic genes *ATF4*, *CHAC1*, *CLIC1* and *PMAIP1* (NOXA) (Fig 4). With regard to ATF4, our data are consistent with previous demonstration that knocking-out *ATF4* renders cells more resistant to mycolactone-induced cell death^4^. Downstream apoptotic targets of ATF4, such as CHOP and BIM, were previously proposed to mediate mycolactone-induced cell death in other, diploid cell lines [4,5,12]. CHOP and BIM were not upregulated by a 24h treatment with mycolactone in KBM-7 cells, suggesting that these events take place at later time points in this experimental setup, or that the pathways connecting ATF4 with apoptosis vary with cell type. In our study, the most upregulated gene was *CHAC1*, which encodes for the glutathione specific gamma-glutamylcyclotransferase 1 [27]. CHAC1 is a sensor of oxidative stress that was reported to mediate cytotoxicity by depleting antioxidative GSH from the cytosol, thereby inducing apoptotic cell death [28]. Interestingly no substantial differences of other key enzymes of the GSH metabolism were found between WT and KO cells (S5 Table). *PMAIP1* (NOXA) belongs to the Bcl-2 family, which mediates programmed cell death by activation of Bak/Bax proteins, and production of ROS at the ER [17]. This pattern of differentially regulated transcripts is therefore in line with previous studies of apoptosis being the main pathway by which mycolactone induces cell death. Our inhibition experiments shown in Fig 6 confirmed that apoptosis is the main cell death pathway induced by mycolactone in KBM-7 cells. Thus, our study identifies a novel, epigenetic mechanism by which SETD1B activates ATF4. In KBM-7 cells, ATF4 upregulation was associated with increased CHAC1 and decreased GSH, suggesting the induction of pro-apoptotic oxidative stress. While this study did not investigate how SETD1B upregulates ATF4, previous work suggest that COMPASS members directly regulate the expression of ER stress mediators [29].

The decrease in cellular GSH that we detected in mycolactone-treated cells supports the concept that reduced GSH levels might be an essential driver of the mycolactone induced apoptotic cell death (Fig 7A). Interestingly, incubation with GSH was found to efficiently raise the intracellular GSH pool, and cells treated with functional GSH were protected from the lethal effects of the toxin (Fig 7A and 7B). However, as mycolactone has an impact on several metabolic pathways, its cytotoxic effect is likely modulated by several factors. Under the given experimental conditions we found a low GSH level as a substantial component resulting cell death of the haploid cell line KBM-7. But mycolactone might have a different impact on other cell lines or tissues.

In summary, the present study is the first to demonstrate the involvement of *SETD1B* in mycolactone-induced cell apoptosis. The results indicate that supplementary treatment of ulcer with GSH might be therapeutically meaningful by reducing the amount of apoptotic cell death thereby decelerating the progression of the disease. An inherent limitation of haploid genetic screens is the failure to detect genes essential for survival such as the main cytosolic target of mycolactone Sec61A, and thus did not emerge in our screen [30]. In addition, the limited availability of mycolactone for large-scale screens does not allow to perform analyses with saturating coverage of the genome. Additional candidates might be identified when screening larger amounts of haploid cells allowing to identify a comprehensive pattern of human genes involved in the toxic effects of the *M*. *ulcerans* macrolide. In addition, we were not able to identify a direct link between SETD1B and the ATF4 pathway within our analysis.

## Methods

### Cell culture

KBM-7 cells were cultured in IMDM supplemented with 10% fetal calf serum FCS (PAN-Biotech), 100 U/ml penicillin, 100 μg/ml streptomycin (PAN-Biotech) and L-glutamine (PAN-Biotech). HEK293T cells (DSMZ, Germany) were grown in DMEM high glucose supplemented with 10% FCS, penicillin (100 U/ml), streptomycin (100 μg/ml), and L-glutamine (PAN-Biotech).

### Insertional mutagenization of KBM-7 cells

The gene-trap retrovirus used for mutagenesis of KBM-7 cells were generated by co-transfecting HEK293T cells with the green fluorescent protein (GFP) carrying gene-trap vector pGT-GFP, in addition to the packaging plasmids pCMV-Gag-Pol (CellBiolabs), pAdVAntage (Promega) and pCMV-VSV-g (CellBiolabs). Transfection of plasmids was done with the cationic liposome lipofectamine 2000 (Invitrogen). After transfection, culture supernatant containing viral particles was collected and concentrated by centrifugation. The retroviral particles carrying a gene trap cassette with an adenoviral splice-acceptor site, a SV40 polyadenylation signal, and a GFP marker gene were used to infect, and mutagenize the haploid KBM-7 cells in the presence on 8 μg/mL protamine sulfate. After infection with the retroviral particles cells were seeded in 24-well plates and centrifuged for 45 min at 2000 rpm. Fluorescence-activated cell sorting (FACS)-based enrichment (FACSAria II system, BD Biosciences) of infected KBM-7 cells were performed to increase the proportion of mutagenized cells. Screens were started with either freshly mutated cells, or stocks of cells frozen at least 3–4 days after mutagenesis.

### Mycolactone screen

Mycolactone used for the experiments was purified from *M*. *ulcerans* bacteria as previously described [3]. The haploid screen was carried out in 96 well plates with 20000 mutagenized cells per well in 200μl IMDM culture medium. Toxicity of mycolactone was tested with the KBM-7 cell line prior to the screening experiments with different concentrations. Incubation of 20nM for three days was found to be lethal for KBM-7 cells. For the screening experiments we thus incubated cells with 20nM mycolactone for at least 3–4 weeks, with renewal of toxin-containing medium every 7 days. KBM-7 wild-type (WT) cells served as controls to verify full toxic activity of mycolactone. Resistant clones were harvested when cells reached a density of 10^7^ cells per well, and DNA was extracted by magnetic beads (LGC-Genomics) for further sequencing analysis.

### Next generation sequencing analysis

Viral integration sites in the human genome were identified by deep sequencing analysis. For next-generation sequencing (NGS) library construction, genomic DNA was digested with either MseI (NEB) or NlaIII (NEB) and ligated with T4 DNA ligase (NEB). Human sequences flanking the viral insertion sites were PCR amplified with primers complementary to the sequence of the viral pGT vector also carrying P5/P7 adaptor for MiSeq (Illumina) sequencing (S6 Table). NGS analysis was performed with a custom sequencing primer. Insertion sites of unselected mutagenized KBM-7 WT cells were amplified by a linear PCR after digestion of DNA with the restriction enzymes NlaIII or MseI. Linear PCR was done with a biotin-coupled primer annealing to the viral insertion site (S6 Table). A dideoxycytidine (ddC)-coupled linker was then ligated to the 3’-end of streptavidin-biotin purified product applying a ssDNA ligase (CircLigase II, Epicenter Biotechnologies). Amplification of host sequences was done with PCR primer complementary to the viral insertion (S6 Table). The purified product was sequenced on an Illumina MiSeq (150 base pair, single end) using the custom sequencing primer to generate reads of the human genome flanking the viral integration sites.

### Bioinformatic analysis

Raw reads of the sequencing analyses were mapped to the human reference genomes hg19 with BOWTIE 2.0 [31]. Only reads uniquely aligning to the human genome were considered for further analyses. Assessment of frequency and location of viral insertions at nucleotide resolution was determined with the HaSAPPy pipeline [32]. Sequence reads obtained from the mycolactone selected cells were compared with those from the unselected control cells to identify genes relevant to the phenotype. As only small numbers of mutated KBM-7 cells (20000 cells/well) were tested in the haploid screen we did not expect to find high numbers of disruptive insertions per gene in our analysis. Rather we assume to find populations of clonally expanded cells with identical insertions. As a consequence, to identify the causal disruptive insertions we considered both the number of in-frame insertions, and the total number of reads per gene, including duplicate insertions of disruptive insertions with HaSAPPy. Insertions with high read numbers located outside genes but in putative gene regulatory sites were also considered. Therefore, intergenic insertions sites with high abundance were screened for regulatory sites with significant effects on gene transcription as found in the eQTLGen database (http://www.eqtlgen.org).

### CRISPR Cas gene editing

Single knockout clones of *SETD1B*, *RELT* and *R3HDM2* were generated with the CRISPR/Cas vector px458 vector containing the EGFP marker (http://www.addgene.org plasmid 48138; Zhang lab). Target sequences for each gene were designed with the CRISPR/guides selection server at crispr.mit.edu (S7 Table). The guide DNA sequences of the three genes were directly ligated into the BbsI restriction site of the px458 vector following the protocol from Zhang et al. (https://www.addgene.org/crispr/zhang/). Gene KOs were generated by transfection of the px458 into the KBM-7 cells. Transfected cells were single-cell sorted in 96-well microtiter plates and then expanded for 2 weeks. DNA and RNA from 10 different KO clones of each of the three genes were sequenced to screen for frame-shift mutations within the coding sequence of the genes (S7 Table). Single KBM-7 knockout clones (20.000 cells) of *SETD1B*, *RELT* and *R3HDM2* where disruptive mutations were verified were incubated with 10nM mycolactone for at least 6 days. To determine the number of surviving cells FACS analysis (LSR II Becton Dickinson) with LIVE/DEAD Fixable Blue Dead Cell (Thermo Fisher Scientific) stained cells counting 10000 events was performed. The number of WT cells and KO clone cells cultured without mycolactone were used as controls.

### Cytotoxic assays

To assess the relevance of apoptosis, necroptosis, and ferroptosis in mycolactone-induced cell death, inhibitors for each pathway were tested. KBM-7 WT cells were seeded at a density of 20,000 cells/well in 96-well flat bottom plates to allow for ~70% confluence on the day of treatment. The inhibitors of programmed cell death were diluted in DMSO before addition to the cells with the exception of ß-mercaptoethanol (ß-ME). Cell death was determined by FACS analysis (LSR II Becton Dickinson) with LIVE/DEAD Fixable Blue Dead Cell (Thermo Fisher Scientific) stained cells counting 10000 events, after 3 days of treatment with 20nM mycolactone. Substances used in this experiment were necrostatin (50μM; Santa Cruz Biotechnology), zVAD-FMK (50μM; Santa Cruz Biotechnology), ferrostatin (1μM; Sigma), liproxstatin (100nM; Sigma), deferoxamine (100μM; Sigma) and ß-ME (50μM; Sigma). Cells were incubated for 3 days with 20nM mycolactone.

### Transcriptome analysis

Total RNA was extracted from KBM-7 cells using Qiazol lysis reagent (Qiagen), then purified using Qiagen RNeasy Mini Kit, and digested with RNase-Free DNase set (Qiagen). For differential transcriptome analysis WT and SETD1B KO KBM-7 cells were incubated with 100nM of mycolactone for 24h a concentration inducing maximal augmentation of the transcript levels of interest. Total RNA was extracted from WT and resistant mutagenized KBM-7 cells treated with and without mycolactone, and reverse transcribed into cDNA. Sequencing was done with 50bp single end reads on an Illumina HiSeq2000 Sequencer. All experiments were performed in triplicates. On average, 30 million reads per experiment were obtained. After quality control, sequencing reads were aligned with HiSAT2 (version 2.1.0) to the human reference genome (GRCh38) [33]. Reads were assigned to genes with the FEATURECOUNTS module of the SUBREAD package (version 1.6.0) [34] based on the Gene transfer Format (GTF) file GRCh38.82. Differential transcriptome analysis was performed with the DESEQ2 software [35] comparing read counts between the groups of WT and *SETD1B* KO treated or not with mycolactone. Normalization of sequencing reads was performed with the DESeq2 median of ratios normalization algorithm. Transcript differences were considered relevant with a log fold change of ≥1.0.

### Validation of differentially expressed genes by RT-PCR

Total RNA from cells was extracted using TRIzol (Thermo Fisher Scientific). 1 μg RNA was used for reverse transcription. 10× diluted cDNA was used for quantitative RT-PCR reactions using the LightCycler 480 System (Roche) and the SYBR Green Master Mix (Roche). All data were normalized to *PPA1* expression. Primer sets for quantitative real-time PCR analyses are shown in S6 Table.

### Glutathione (GSH) and N-acetylcysteine (NAC) supplementation

To investigate whether supplementation of cells with external GSH or NAC increase intracellular GSH levels we incubated KBM-7 cells and *SETD1B* KO cells with the corresponding antioxidants. Total GSH levels were measured with a glutathione ELISA kit (Abnova). Survival analyses were performed with LIVE/DEAD stained cells incubated with 10 or 20mM GSH (Sigma) or with 10mM NAC (Sigma).

### Statistical analysis

The results are expressed as the mean ± standard deviation and were calculated from quantitative data obtained from replicate experiments. Statistical significance of the differences between two groups was determined using t-test unless otherwise stated. The p-values ≤ 0.05 were considered significant calculated with Stata 14.0. Statistical significance was determined by analyzing the data with the parametric test.

## Supporting information

S1 Table Genes identified by NGS analysis of the mycolactone resistant clone 1. (XLSX)

S2 Table Genes identified by NGS analysis of the mycolactone resistant clone 2. (XLSX)

S3 Table Differentially regulated genes of WT and KO KBM7 cells treated or not with mycolactone. (XLSX)

S4 Table Gene ontology analysis. (XLSX)

S5 Table Expression of genes involved in the glutathione metabolism. (XLSX)

S6 Table Primer and adapter for NGS analyses of mycolactone selected cells and unselected controls. (XLSX)

S7 Table Primer used for CRISPR/Cas KO and sequencing analyses. (XLSX)
